# Advancing Tau-PET quantification in Alzheimer’s disease with machine learning: introducing THETA, a novel tau summary measure

**DOI:** 10.21203/rs.3.rs-3290598/v1

**Published:** 2023-10-18

**Authors:** Robel K. Gebre, Alexis Moscoso Rial, Sheelakumari Raghavan, Heather J. Wiste, Kohl L Johnson Sparrman, Fiona Heeman, Alejandro Costoya-Sánchez, Christopher G. Schwarz, Anthony J. Spychalla, Val J. Lowe, Jonathan Graff-Radford, David S. Knopman, Ronald C. Petersen, Michael Schöll, Clifford R. Jack, Prashanthi Vemuri, Alzheimer’s Disease Neuroimaging Initiative

**Affiliations:** 1 Department of Radiology, Mayo Clinic, Rochester, MN 55905, USA; 2 Department of Psychiatry and Neurochemistry, Institute of Neuroscience and Physiology, The Sahlgrenska Academy, University of Gothenburg, Gothenburg, Sweden; 3 Wallenberg Centre for Molecular and Translational Medicine, University of Gothenburg, Gothenburg, Sweden; 4 Universidade de Santiago de Compostela, Santiago de Compostela, Spain.; 5 Centro de Investigación Biomédica en Red sobre Enfermedades Neurodegenerativas (CIBERNED), Instituto de Salud Carlos III, Madrid, Spain.; 6 Nuclear Medicine Department and Molecular Imaging Group, Instituto de Investigación Sanitaria de Santiago de Compostela (IDIS), Travesía da Choupana s/n, Santiago de Compostela, 15706, Spain.; 7 Department of Qualitative Health Sciences, Mayo Clinic, Rochester, MN, USA; 8 Department of Neurology, Mayo Clinic, Rochester, MN 55905, USA; 9 Dementia Research Centre, Queen Square Institute of Neurology, University College London, UK

## Abstract

Alzheimer’s disease (AD) exhibits spatially heterogeneous 3R/4R tau pathology distributions across participants, making it a challenge to quantify extent of tau deposition. Utilizing Tau-PET from three independent cohorts, we trained and validated a machine learning model to identify visually positive Tau-PET scans from regional SUVR values and developed a novel summary measure, THETA, that accounts for heterogeneity in tau deposition. The model for identification of tau positivity achieved a balanced test accuracy of 95% and accuracy of ≥87% on the validation datasets. THETA captured heterogeneity of tau deposition, had better association with clinical measures, and corresponded better with visual assessments in comparison with the temporal meta-region-of-interest Tau-PET quantification methods. Our novel approach aids in identification of positive Tau-PET scans and provides a quantitative summary measure, THETA, that effectively captures the heterogeneous tau deposition seen in AD. The application of THETA for quantifying Tau-PET in AD exhibits great potential.

## 1. Introduction

Alzheimer’s disease (AD) is characterized by the accumulation of β-amyloid (Aβ) plaques and neurofibrillary tangles (NFTs) in the brain. NFTs are composed of hyperphosphorylated tau proteins and in a majority of individuals tau progresses along predictable patterns, originating in the transentorhinal cortex and spreading to the limbic system and eventually to the neocortex. The spread of tau leads to cognitive impairment and dementia^1^. However, evidence from pathology and imaging have shed light on the heterogeneity of tau deposition in AD, suggesting that there could be distinct patterns of tau accumulation across individuals^2–4^.

Current understanding of AD pathophysiology and neurodegeneration suggests that the NFT accumulation is closely correlated with clinical disease progression and precedes clinical symptoms, making tau a promising biomarker for disease diagnosis and clinical trial design^5,6^. Positron emission tomography (PET) imaging is used to visualize and assess tau deposition using radioligands that bind specifically to the paired helical filament of NFTs and can be used to detect and track tau pathology in vivo^7^. Studies using PET have shown in preclinical AD, tau deposition is spread throughout several cortical regions and there follows multiple trajectories^3^. The most common quantification methods for Tau-PET utilize meta-regions of interest (meta-ROIs), such as the temporal meta-ROI, or the more recent medial temporal lobe (MTL) and neocortical (NEO) meta-ROIs to stage disease severity^8,9^. These methods ignore the extent of tau outside these meta-ROIs and average the Tau-PET standardized uptake value ratios (SUVR) in the entire meta-ROI, which underweights any focal depositions of tau in smaller regions within the meta-ROI. In addition to the meta-ROIs, there are less commonly used quantitative methods such as the volumes-of-interest voxel-based multiblock barycentric discriminant analysis (MUBADA)^10^ that have also been used to assess the clinical group separation.

The visual rating method followed in this study was based on the density and distribution of tau identified by the radiotracer [^18^F]flortaucipir (Tauvid^™^) which was recently FDA-approved for AD tau pathology at B3-level (Braak stages V/VI)^11^. The visual assessment criteria consider the focal deposition of tau through the brain and could overcome the limitations of the meta-ROI methods. In this work we set out to test the hypothesis that a machine learning (ML) model can be developed to identify positive Tau-PET scans based on the clinically accepted multirater visual ratings, and improved quantification methods can be developed to incorporate the heterogeneity in spatial distribution of tau tracer signals throughout the brain. We further hypothesized that these ML-based tau quantification methods could outperform the currently used meta-ROI quantification methods and provide a more accurate and sensitive quantification of tau deposition that would map better to disease severity.

To test our hypotheses, we designed our study with three aims: 1) develop a machine learning model on a large single site dataset using regional SUVR values as inputs and visual ratings as targets and validate the model’s performance on two external independent cohorts, 2) compare the performance of our ML model to temporal, MTL and NEO meta-ROI quantitative methods, and 3) develop a novel summary measure that is more sensitive to clinical disease severity by leveraging the regional heterogeneity captured by our ML model. This study aims to address the limitations in the current quantitative methods for tau deposition in AD by utilizing advanced ML approaches.

## 2. Results

### 2.1. Characteristics of study population

The study included three independent datasets: Mayo, ADNI, and OASIS-3. The Mayo dataset had 1290 participants with an average age (SD) of 67 (14) years: 55% were male, and 74% were cognitively unimpaired. The ADNI dataset had 831 participants with an average age of 72 (8) years: 48% were male, and 55% were cognitively unimpaired. The OASIS-3 dataset had 430 participants with an average age of 70 (8) years: 43% were male, and 86% were cognitively unimpaired (Table 1). The percentage of visually tau-positive cases in Mayo, ADNI, and OASIS-3 were 19%, 28%, and 14%, respectively (Table 1). The proportion of participants who were classified as tau-positive using both MTL and NEO meta-ROIs were low, highlighting the heterogeneity of the sample (14% for Mayo, 20% for ADNI, and 11% for OASIS-3) (Table 1).

### 2.2. Model trained on visual ratings for predicting tau positivity

**Figure 1.**
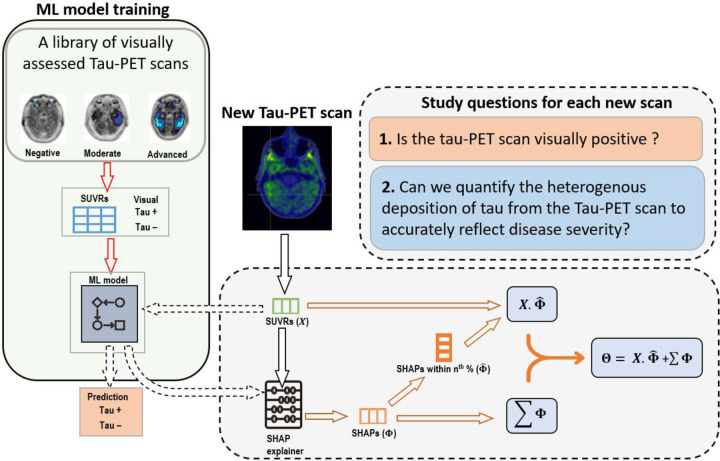
Study design. First, we trained a machine learning (ML) model using a library of visually assessed scans where the visual rating was used as the ground truth and the SUVRs were the inputs. Second, after training the model we applied the SHAP AI explainer to determine each region’s contribution to the predicted visual rating. Lastly, we derived a summary measure we are calling tau heterogeneity evaluation in Alzheimer’s disease (THETA) score using each participant’s SUVR value and corresponding SHAPs.

**Figure 2.**
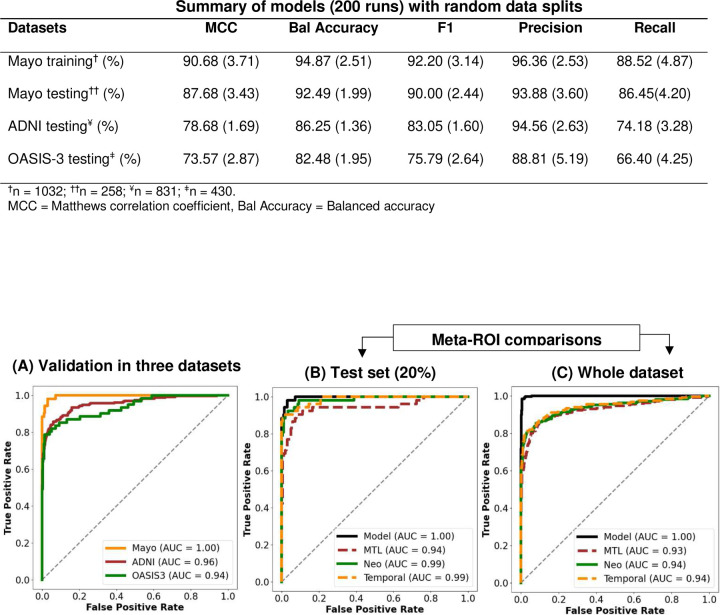
Model performance for binary classification of tau status based on the visual assessment from the three raters. The model was trained on the Mayo and validated on the external validation sets, ADNI and OASIS-3. The top table shows summary of the multiple runs conducted using different random splits of the training (80%) and testing (20%) sets. The metrics in the table show the mean (standard deviation). The receiver operating characteristic’s area under the curve (AUC) of the model (A) compares its performance in Mayo, ADNI, and OASIS3, while (B) and (C) illustrate the comparison of the model’s performance to meta-ROI classification schemes in the Mayo testing and whole dataset respectively.

The regional SUVRs were the inputs to the ML model and the visual classifications were the predicted class (Fig. 1). The model was trained on the Mayo dataset and tested on ADNI and OASIS-3. To validate the model, we conducted multiple runs using different data splits (Fig. 2). The models’ performance was consistent as indicated by a standard deviation less than 5% for all metrics (Fig. 2). We then selected the best model with the highest f1-score.

The best model performed very well in predicting tau status on the Mayo dataset, achieving a balanced accuracy of 98.58% and 95.43% on the Mayo training and testing sets, respectively. When evaluating the model’s performance on the external datasets, ADNI and OASIS-3, it achieved a balanced accuracy of 87.74% and 87.03%, respectively. The model identified tau-positive and negative participants with an AUC of 1.00 on the testing set. It also showed very good classification performance on the ADNI external dataset, with an AUC of 0.96. In contrast, the AUC was lower in the OASIS-3 dataset at 0.94 (Fig.2).

### 2.3. Model performance in comparison to meta-ROI-based assessment for prediction of tau positivity

The meta-ROIs showed very similar performances in classifying tau positivity in the Mayo cohort, with an AUC of 0.99 on the test-set (20%) and 0.94 on the whole dataset (Fig. 2B and Fig. 2C). The model outperformed all three meta-ROIs when evaluating classification performance on the Mayo dataset, with a misclassification of 3.67% and 0.48% of tau-positive and negative cases, respectively. On the ADNI dataset, the model misclassified 22.17% of the tau-positive and 2.33% of the tau-negative cases and was largely outperformed by the temporal meta-ROI for tau-positive misclassification at a rate of 6.96% (Table 2). On the OASIS-3 dataset, the model performed best in classifying tau-negative cases with a misclassification rate of 1.36% and had the second-best misclassification rate of 24.59%, outperformed by the temporal meta-ROI at 18.03%. *Supplementary Tables 1 and 2*provide similar analyses for participants with CI and CU clinical diagnosis.

### 2.4 Spatial heterogeneity captured by the machine learning model

**Figure 3.**
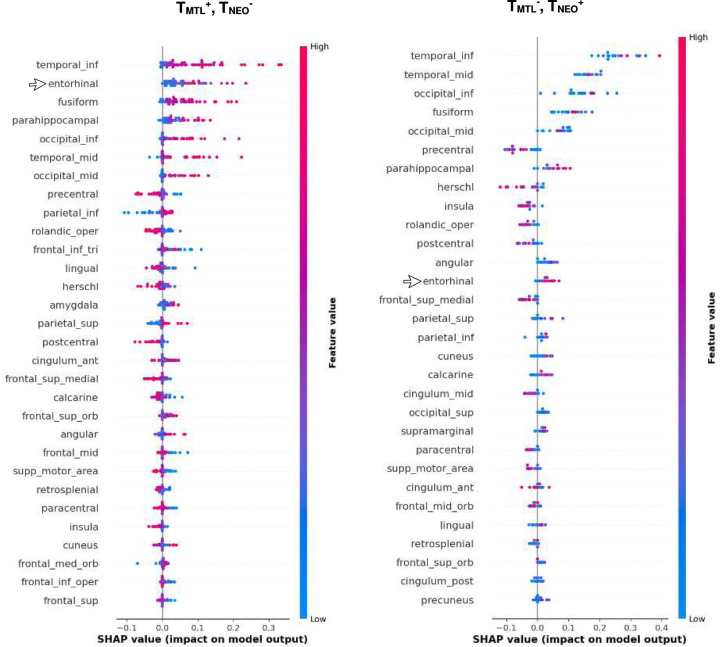
Feature importances for cases where tau was positive in the MTL meta-ROI only and in NEO meta-ROI only. The arrow indicates the importance of the entorhinal region changing its rank depending on the regionality for T_MTL_^+^, T_NEO_^−^ (left) cases, and for T_MTL_^−^, T_NEO_^+^ (right).

To assess the spatial heterogeneity captured by the model, we analyzed the SHAP (SHapley Additive exPlanations)^12^ summary plots for tau in the different regions of the brain. In participants with tau positivity in the NEO region, the inferior temporal cortex region was the top predictor (Fig.3). Conversely, in participants with tau positivity in the MTL region (the region well-known to be affected by tau deposition), the entorhinal cortex region emerged a crucial predictor (Fig. 3).

### 2.5. Novel tau summary measure – THETA score

We designed a novel tau global summary measure, THETA score (Tau Heterogeneity Evaluation in Alzheimer’s Disease), that considers the spatial heterogeneity of tau deposition throughout the brain.

**Figure 4.**
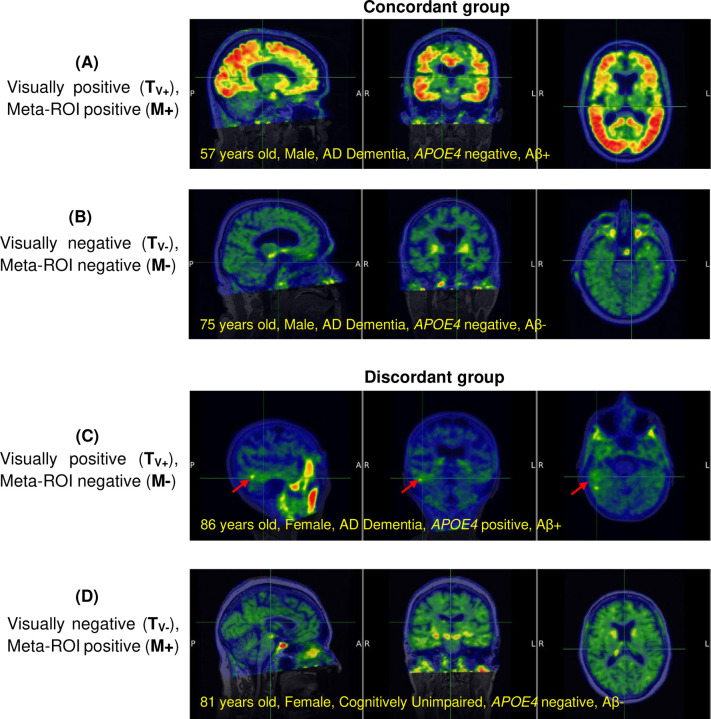
Examples of the concordant groups (A and B) where there is agreement between the visual rating and all three meta-ROIs while the discordant groups (C and D) have disagreement visually and with all three meta-ROIs. While the meta-ROI can miss visually positive scans where the SUVR is lower than the cutoff point (C), the visual assessment does not consider isolated increased activity in the MTL (D). The red arrows indicate where there is increased tracer uptake activity.

The THETA score considers the contribution of all the regional tau SUVRs used to the determine a tau-positive or tau-negative scan. Here we illustrate THETA in two sub-populations that highlight tau heterogeneity: discordant and concordant groups. The discordant group consist of cases where there is disagreement between the visual rating and one or more of the meta-ROI classifications while concordant group consists of cases that agree both visual and with the meta-ROIs (Fig. 4).

**Figure 5.**
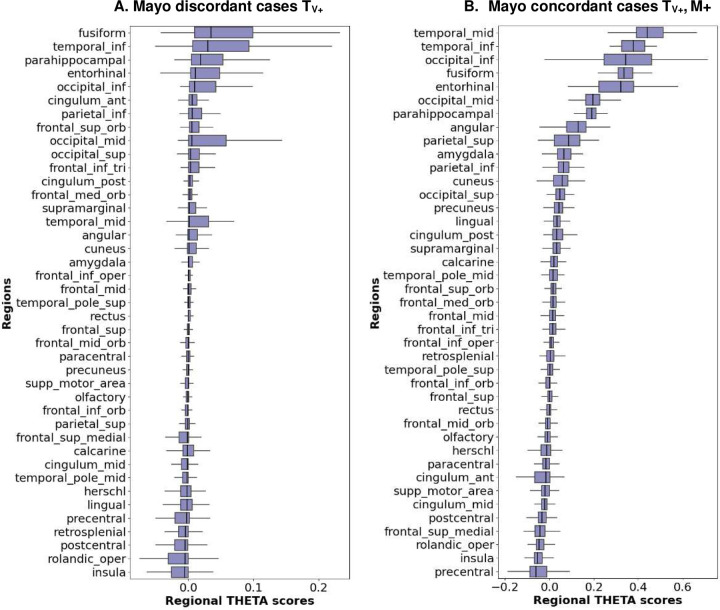
The average regional THETA scores ranked in ascending order by median value for discordant cases (left) and concordant cases (right). The discordant cases which were visually positive (T_V+_) and negative with one or more meta-ROIs, and the concordant cases which were tau-positive (T_V+_ M_+_) both visually and all three meta-ROIs.

The THETA score, as described in Equation 2 (section 4.6), was developed to combine different regions based on their contribution to both classification and disease severity, as indicated by the SUVRs. In the tau-positive and meta-ROI negative discordant cases where the model contribution is distributed amongst different regions and not focused specifically on meta-ROI regions, the THETA formulation successfully captures the heterogenous contributions of all the regions, including those with relatively mild signals and similar contributions (Fig. 5A). On the other hand, in tau-positive concordant cases, the hotspot regions that constitute the meta-ROIs are the top predictors in our ML model. In these cases, the THETA formulation maintains the importance of the top regions, thereby preserving the spatial heterogeneity (Fig. 5B).

#### 2.5.1. Performance of THETA for assessing disease severity

The performance of the tau summary score THETA for disease severity was assessed using two clinical disease severity measures, Mini-Mental State Examination (MMSE) and CDR sum of boxes (CDR-SB).

**Figure 6.**
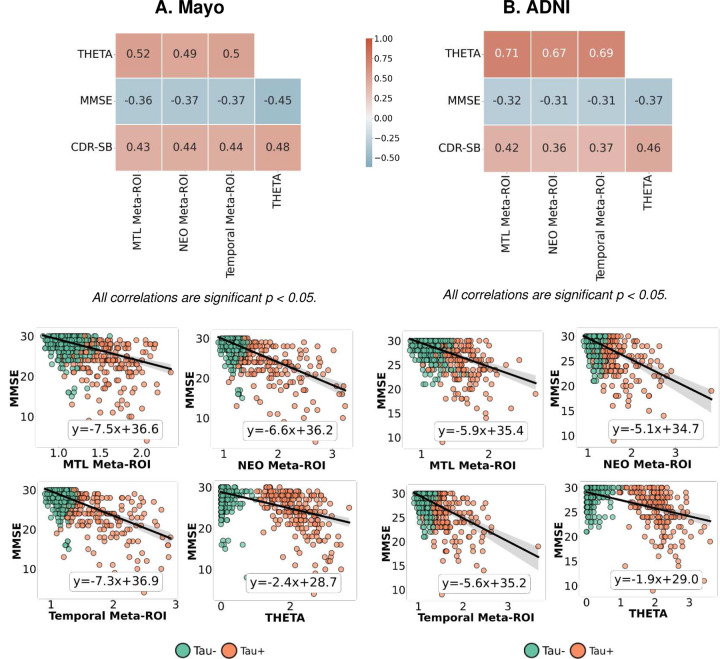
Comparison of the meta-ROIs and THETA score to the clinical measures MMSE and CDR-SB. The correlation coefficients are Spearman’s *rho* and the scatter plot shows the ordinary least squares regression. Similar results for the OASIS-3 cohort are included in *Supplementary Figure 2*. Tau− and Tau+ labels indicate visual assessment status.

**Figure 7.**
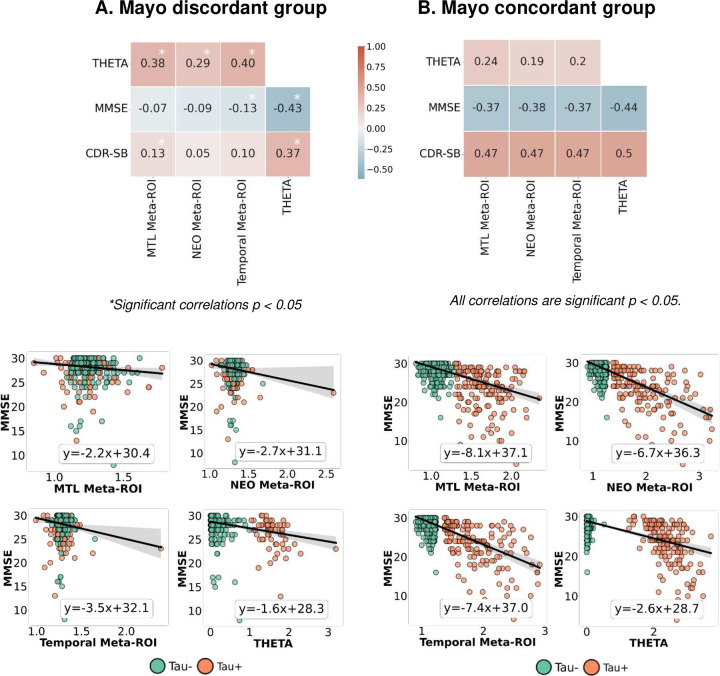
Comparison of the meta-ROIs and THETA to clinical scores MMSE and CDR-SB for the Mayo cohort in the discordant and concordant group. The discordant group consisted of participants with disagreement between the visual rating and one or more meta-ROIs on the tau status, and the concordant group consists of participants whose tau status had agreement between the visual and all three meta-ROI methods. A similar analysis with outliers removed is included in *Supplementary Figure 3*. Tau− and Tau+ labels indicate visual assessment status.

When correlation was conducted for all participants from each cohort, the performance of the THETA score and the meta-ROIs was similar (Fig. 6, OASIS-3 shown in *Supplementary Fig. 2*). When looking at the relationship of MMSE to the meta-ROIs and THETA, there was a similar trend of decreasing slope from tau-negative to tau-positive (Fig. 6). However, the THETA score provided a clearer and more distinct separation between tau-positive and negative participants (Fig. 6). This pattern was also observed in the concordant groups (Fig. 7). In contrast, for the discordant groups, THETA demonstrated a negative and significant association with MMSE and a strong positive association to CDR-SB, but the meta-ROIs were not significantly associated with MMSE (Fig. 7). Similar analysis with possible outliers excluded is shown in *Supplementary Figure 3*.

**Figure 8.**
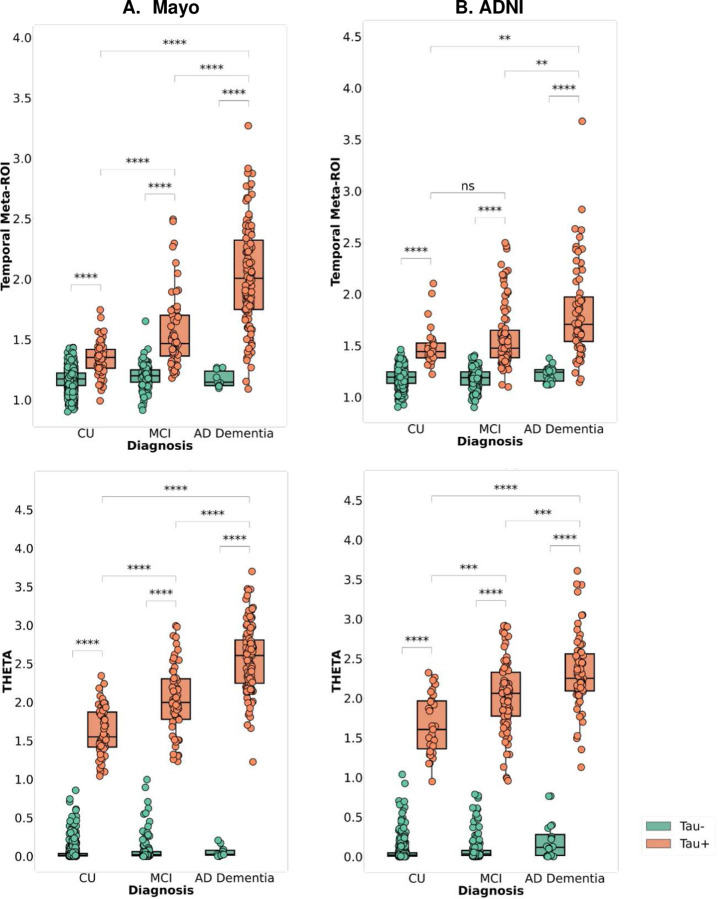
Comparison of the distribution of temporal meta-ROI and THETA in diagnostic groups for visually tau positive and negative participants. Mayo participants and on the right the ADNI participants are shown on the left and ADNI participants on the right. Tau− and Tau+ labels indicate visual assessment status. ns: p <= 1.00, *: 0.001 < p <= 0.005, **: 0.0001 < p <= 0.001, ***: 0.00001 < p <= 0.0001, ****: p <= 0.00001

Furthermore, we compared THETA to the temporal meta-ROI for different clinical diagnostic outcomes and calculated the mean differences between tau-positive and tau-negative cases (Fig. 8). We found that for the AD Dementia participants the separation between the tau-positive and tau-negative cases created by both temporal Meta-ROI and THETA were similar in terms of statistical significance across the disease groups. However, for CU and MCI participants there was a clear overlap in tau status for the temporal Meta-ROI, whereas the THETA score showed better separation between tau-positive and tau-negative cases (Fig. 8). For instance, in the ADNI cohort, the difference between the tau-positive and tau-negative temporal Meta-ROI values for CU and MCI participants had an effect size of 3.08 (t-statistics = 16.50, *p* < 0.001) and 2.23 (t-statistics = 16.76, *p* < 0.001), respectively. In contrast, the THETA score showed a much larger effect size of 10.09 (t-statistics = 54.09, *p* < 0.001) and 6.83 (51.36, *p* < 0.001), respectively (Fig. 8).

## 3. Discussion

The progression of tau pathology, as captured by Tau-PET scans, has become a key indicator of disease severity in AD. However, current methods have limitations in addressing the heterogeneity of tau deposition. They focus on a limited number of regions with typically high tau uptake while ignoring the spatial variance of tau burden within these regions. These two limitations hamper the performance of meta-ROI-based methods for accurate detection and quantification of the Tau-PET signal. Using visual assessment by three raters as the gold standard in a large single site dataset (Mayo), we developed a ML model to accurately classify the status of Tau-PET scans and validated it in two independent datasets (ADNI and OASIS-3). We then utilized the model to develop a novel tau summary measure that considers tau SUVRs across the brain and provides a metric that maps extremely well to disease progression compared to current methods.

### Identification of positive Tau-PET scans

The application of deep learning and ML using Tau-PET has become common in recent years, either to improve PET image acquisition^13^, to classify spatial patterns^14,15^, to study the association between Aβ and Tau-PET scans^16^, or to predict pathological tau accumulation from clinical measures^17,18^. ML-based indices have also been introduced such as Spatial Pattern of Abnormality for Recognition of Early Tauopathy (SPARE-Tau)^19^ and Alzheimer’s disease resemblance atrophy index (AD-RAI)^20^. SPARE-Tau was trained on tau SUVRs to predict clinical status (CU vs MCI/AD) while AD-RAI was trained on T1-weighted MRI volumetric measures also to predict clinical status and quantify brain atrophy. Nonetheless, our work is the first to develop and validate a ML model to identify positive Tau-PET scans using regional SUVRs from the entire brain. We validated our ML model with entirely independent datasets comprised of different population demographics and data sources. More importantly, our model was able to generalize to both multicenter and single-center studies, with ADNI being a multicenter study while OASIS-3 is a single-center study.

Multirater visual assessment of Tau-PET is a clinically accepted standard for identifying positive Tau-PET scans as it offers the possibility of assessing tau burden in the entire brain. It can be superior to the meta-ROIs quantitative methods that rely on specific regions to quantify tau burden. While the meta-ROIs focus on the entorhinal cortex and tend to overestimate tau-positive cases, the visual assessment does not consider isolated tau deposition in the medial temporal lobe. The NEO meta-ROI’s true negative rate was consistent across all three datasets while the MTL did better at identifying true negatives in Mayo and decreased in performance in ADNI and OASIS-3. On the other hand, in the Mayo cohort, all three meta-ROIs underperformed when identifying tau-positive cases compared to the visual ratings. Nonetheless, because our model was trained on the visual ratings, it showed excellent agreement with the visual ratings in the Mayo cohort.

### Quantification of heterogeneity of Tau-PET signal: THETA score

Prior works have shown the spread of tau pathology to be heterogenous and to follow specific patterns across the brain. A histological study by Murray et al. has shown clinical differences between hippocampal sparing and limbic-predominant AD subtypes^21^ while a recent event-based computational study by Vogel et al. has shown the presence of posterior and lateral temporal subtypes of atypical AD^2^. While heterogeneity in tau deposition is accepted in the field, there are no measures that consider the heterogeneity in the Tau-PET signal while quantifying it into a summary metric.

In this study, the novel tau summary measure, THETA, considers spatial heterogeneity across the brain, making it a better option for cognitive assessment and clinical diagnosis. Since the ML model accurately classified Tau-PET scans as tau-positive or negative by examining signals throughout the entire brain, we incorporated the THETAi values to formulate our summary measure. This measure was derived using SHAP values, which indicated the importance of each individual region. Thus, by utilizing the heterogeneity captured by the ML model, we were able to ensure that THETA captured pattern-based information. This is illustrated by the regional THETA scores for the concordant or discordant subgroups (Fig. 3 and Fig. 5). Furthermore, since the range of THETA scores were distinct for the tau-positive/negative cases, we were able to get a clear separation between the tau-positive and negative participants for the MMSE clinical score (Fig. 6 and Fig. 7) and the diagnostic groups better than the temporal Meta-ROI (Fig. 8).

### THETA score for assessing disease severity

Tau is a proximal surrogate of clinical disease severity and Tau-PET has tremendous potential to significantly impact clinical practice and clinical trials. The FDA approved [18F] flortaucipir PET imaging for detecting NFT B3 corresponding to Braak stages V or IV. Hence, effectively quantifying the Tau-PET signal has important implications because it provides a more accurate and sensitive assessment of disease severity. Given that multirater visual assessment is the clinically accepted standard in the field, developing a highly accurate model using this gold standard and utilizing the model characteristics for quantification of Tau-PET signal has several advantages. This is reflected in the THETA score outperforming the current methods as observed in Figures 6 – 8. Additionally, the THETA scores mapped on to cognitive indices comparably or better than meta-ROI-based methods.

THETA can be utilized with ease across multiple clinical studies. The calculation of THETA in a clinical or research setting is similar to the meta-ROI calculation. Once an ML model is trained on the regional SUVRs and is interpreted using the SHAP AI explainer, THETA scores can be generated automatically using our formula. This process can be done for a single participant or a list of participants. The training of a ML model need only be done once and the trained model can be used multiple times, and the training set can constitute cohorts of different demographics as we have demonstrated in our study. Future work will focus on validating THETA for tracking longitudinal Tau-PET changes.

### Strengths and Limitations

This study has some strengths and limitations. We developed a ML model on one dataset and validated it on two independent datasets. There were some limitations in this study. First, the visual assessment of scans is subjective and can be prone to human errors. However, the visual ratings were obtained independently from three raters, and ambiguous discordant cases were reassessed by a Neuroradiologist (CRJ). Second, as expected, the model’s performance was lower for the ADNI, and OASIS-3 validation sets due to differences between the cohorts. However, combining the cohorts and training a new model on the combined data solved this problem. The combined model achieved balanced accuracy between 94% and 96%, and ROCAUC greater than 0.99 for all datasets. This is shown in *Supplementary Table 3* and *Supplementary Figure 4*. Third, the THETA score exhibits high sensitivity for a given tau-status which can be strength or a limitation. While visually accurate classification can provide a better range for tau quantification, a visually inaccurate classification (< 1% cases) could force the THETA towards zero. Future studies are planned to validate its performance on longitudinal studies. Lastly, changing of the cut-points for the meta-ROIs than ones used in this study could change the results for the meta-ROI comparisons.

In conclusion, this study aimed to address the limitations of the current quantitative methods for quantifying the spread of tau deposition in Alzheimer’s disease by using advanced ML approaches. We also developed a novel summary measure that captures regional heterogeneity, which can be a useful clinical tool for assessing disease progression and subtypes and identifying potential therapeutic targets. Further studies are needed to test the versatility of THETA. The ML model developed in this study performed extremely well in predicting tau status on both the MAYO dataset as well as on the external datasets. The model outperformed the three meta-ROIs in classifying tau positivity on the Mayo test set and was comparable in ADNI and OASIS-3. Additionally, the novel summary measure, THETA, was able to better quantify the spatial heterogeneity of tau deposition and provide a more sensitive measure of clinical disease severity. Overall, the study provides promising results for the use of ML models in improving the detection and quantification of tau pathology in Alzheimer’s disease.

## 4. Methods

### 4.1. Study participants

We included participants who had undergone a Tau-PET scan with [^18^F]flortaucipir tracer from three studies: a combined Mayo Clinic Study of Aging (MCSA)^22^ and Mayo Alzheimer’s Disease Research Center (ADRC) data set (N = 1290, referred to as Mayo), Alzheimer’s Disease Neuroimaging Initiative phase 2 or 3 (ADNI) (N = 831), and Open Access Series of Imaging Studies phase 3 OASIS-3 (N = 430)^23^. Individuals with frontotemporal dementia were excluded. The Mayo cohort is a population-based study of cognitive aging among residents of Olmsted County, Minnesota, while the ADRC is a longitudinal research study of individuals recruited from clinical practice, and all participants provided written informed consent. Both studies have been approved by the Mayo Clinic and Olmsted Medical Center Institutional Review Boards. The ADNI cohort initiative was as launched in 2003 as a public-private partnership, led by Principal Investigator Michael W. Weiner, MD. The primary goal of ADNI has been to test whether serial magnetic resonance imaging (MRI), positron emission tomography (PET), other biological markers, and clinical and neuropsychological assessment can be combined to measure the progression of mild cognitive impairment (MCI) and early Alzheimer’s disease (AD). The ADNI data was obtained from adni.loni.usc.edu database and for up-to-date information, see www.adni-info.org. The OASIS-3 cohort is a longitudinal study through WUSTL Knight ADRC’s ongoing projects including cognitively normal adults and individuals at various stages of cognitive decline, with MR and PET scans available. The data was obtained through request at https://www.oasis-brains.org/.

### 4.2. Image Preprocessing and SUVR measurements

T1-weighted MRI were tissue-class segmented and divided into atlas regions using the MCALT-ADIR122 atlas^24^. Tau-PET scans were rigidly coregistered to corresponding MRI and median values were taken for each region. Cortical and subcortical regions were referenced to the median of the cerebellar crus to form SUVR units. These regional SUVR values were used both to form the meta-ROIs and as inputs to our machine learning models (see Section 4.5).

### 4.3. Visual assessment of Tau-PET scans

We followed the FDA-approved official criteria for visual assessment to classify the scans in the study^11,25^. In addition, the visual assessment on Tau-PET scans in all data sets was performed independently by three trained raters. Readers examined the PET images scaled to the average counts in a 2D cerebellum ROI and assigned either a positive (increased neocortical tracer uptake isolated to the posterolateral temporal or occipital or parietal/precuneus regions with or without frontal activity) or negative (no increased neocortical activity or increased neocortical activity isolated to the mesial temporal, anterolateral temporal, and/or frontal regions) AD pattern status using a previously published visual interpretation method^25^ (*Supplementary Fig. 5*).

### 4.4. Tau-PET status using meta-ROIs

The temporal meta-ROI was a voxel-weighted average of median uptake in the entorhinal, amygdala, parahippocampal, fusiform, inferior temporal, and middle temporal regions with the cerebral crus gray median as a reference region^8^. A cutoff point of 1.23 SUVR was used to assess tau positivity for the temporal meta-ROI. The MTL was an unweighted average of medial Tau-PET uptake in bilateral entorhinal cortex and amygdala while the NEO meta-ROI was a voxel-weighted average of bilateral middle temporal and inferior temporal gyri^9^. Meta-ROI values above 1.30 SUVR for MTL and above 1.73 for NEO were considered abnormal.

### 4.5. Training and interpreting the machine learning model

The inputs to the ML model were 41 cortical region SUVR values calculated as mean of the right and left hemispheres values. The final model was trained on the Mayo dataset (n = 1290) split into 80% training (n = 1038) and 20% testing (n = 252) and was evaluated on the external datasets ADNI (n = 831) and OASIS-3 (n = 430). To validate the effect of the data splitting on the model performance, we split the data using 200 random seeds and ran the models on the different partitions (*Supplementary Fig. 1*). To account for class (tau-positive vs tau-negative) and group imbalance (discordant vs concordant) we implemented a semi-random iterative stratified data splitting technique (*Supplementary Fig. 6*).

We used a multi-layer stack ensemble machine learning technique with a repeated k-fold bagging to train our model. Repeated k-fold bagging randomly partitions the training data into k folds and then trains k models, each using a different fold as the validation set. This process is repeatedly cross validated with the folds changing each time. The final ensemble model is then created by averaging the predictions of the k models. The Autogluon package was used for this purpose^26^. We preferred this technique due to its robustness and less likelihood of overfitting^26^.

In order to interpret the model we used SHAP (SHapley Additive exPlanations)^12^. SHAP is a model-agnostic approach to interpreting model predictions that assigns a value to each feature which indicates how much a feature has contributed to the final prediction^12^. To develop the new metric THETA (Section 4.5), we made use of SHAP’s Associative property, which states that the individual contributions sum up to the target label. In our binary problem of tau positivity, the SHAP values for each region ranged between −1 and +1 and for each tau-PET scan’s regional SUVR values these SHAPs added up to either a 0 (tau-negative) or +1 (tau-positive).

### 4.6. Developing the novel tau summary measure

We have developed a novel summary measure which we termed as the THETA score (Tau Heterogeneity Evaluation in Alzheimer’s Disease). This score is calculated as a linear combination of two components: the model outputs based on the contributions of tau SUVRs across the entire brain, and the weighted contribution of the SUVRs that fall within the 1^st^ and the 99^th^ percentile of SHAP values (Equation 1). The first component captures the overall feature importance by summing the SHAP values $\left(\Sigma_{i=1}^{m}\phi_{i}\right)$ across *m* number of regions. These SHAP values represent the individual contribution of each brain region (*i*) to the model’s prediction. The second component $\left(\Sigma_{i=1}^{m}{\hat{\phi }}_{i}x_{i}\right)$ focuses on the weighted contribution of the brain regions whose SHAP values fall within the percentile range. Across this subset, the SHAP values $\left({\hat{\phi }}_{i}\right)$ and the actual values of the corresponding SUVRs (*x*_*i*_) are multiplied to reflect their scaled impact. By combining these two components, the THETA score provides a comprehensive assessment of tau accumulation over the whole brain.


(1) $$\Theta =\sum_{i=1}^{m}\phi_{i}+\sum_{i=1}^{m}{\hat{\phi }}_{i}x_{i}$$

Where φ_*i*_ are SHAP values, ${\hat{\phi }}_{i}$ are the SHAP values within the percentile range, *x*_*i*_ are the corresponding regional SUVRs, and *m* is the total number of brain regions.

To assess the repeatability of the THETA scores, we calculated the intra-class correlation coefficient (ICC) of the top models. We found the smallest ICC was 0.97 and the largest ICC was 1.00 (*Supplementary Fig. 1*).

### 4.7. Statistical Analysis

Model performance was evaluated using Mathews correlation coefficient, balanced accuracy, precision, recall, and F1-score. Classification performance of the model and the meta-ROIs was measured on the test-set using Receiver Operating Characteristics Area Under the Curve (ROC AUC). The predicted probabilities and the raw SUVRs were used to plot the ROC AUC curve for the model and meta-ROIs, respectively. To compare the visual assessments to the meta-ROIs or to the ML model’s predictions, we used the true positive rate (TPR = TP / (TP + FN)), which is also known as sensitivity, true negative rate (TNR = TN / (TN + FP)), also known as specificity, rate of tau-positive mismatch (1-TPR), and rate of tau negative mismatch (1-TNR). In addition, we evaluated the performance of THETA on the clinical disease severity measures by calculating correlation using Spearman *rho* and a linear estimation of slope and intercept using ordinary least squares. Lastly, we evaluated the separation between tau-positive and tau-negative for the different clinical diagnosis groups using Cohen’s *d* for effect size and performed mean comparison using two-tailed independent samples t-test with Bonferroni correction for multiple comparisons.

## Figures and Tables

**Table 1.** Characteristics summary of study population.

Variables	Mayo	ADNI	OASIS-3
N	1290	831	430
Age, *mean (SD) years*	67 (14)	72 (8)	70 (8)
Males, n (%)	706 (55)	399 (48)	186 (43)
Females, n (%)	584 (45)	432 (52)	244 (57)
Cognitively unimpaired (CU), n (%)	957 (74)	455 (55)	371 (86)
Mild cognitively unimpaired (MCI), n (%)	173 (13)	283 (34)	11 (3)
Alzheimer’s disease (AD), n (%)	121 (9)	93 (11)	48 (11)
Dementia with Lewy Bodies (DLB), n (%)	37 (3)	-	-
*APOE4*+, n (%)	425 (34)	287 (40)	168 (39)
Aβ+, n (%)	512 (40)	335 (43)	133 (32)
T_v+_, n (%)	245 (19)	230 (28)	61 (14)
T_MTL_^+^, n (%)	243 (19)	255 (31)	80 (19)
T_NEO_^+^, n (%)	202 (16)	183 (22)	57 (13)
T_Temporal_^+^, n (%)	476 (37)	418 (50)	159 (37)
T_MTL_^+^ and T_NEO_^+^, n (%)	183 (14)	170 (20)	49 (11)
T_Temporal_^+^ and T_MTL_^+^, n (%)	235 (18)	242 (29)	74 (17)
T_Temporal_^+^ and T_NEO_^+^, n (%)	202 (16)	183 (22)	57 (13)

T_V+_: Visually tau-positive

T_Temporal_^+^: Tau-positive in the temporal meta-ROI

T_MTL_^+^: Tau-positive in the middle temporo-lateral (MTL)

T_NEO_^+^: Tau-positive in the neocortex (NEO)

**Table 2.** Comparison of Meta-ROI-based assessments and the machine learning model predictions to the visual ratings when predicting tau positivity.

Comparisons	TPR (%)	TNR (%)	TP (n)	TN (n)	1 - TPR (%)	1 - TNR (%)
MAYO^†^						
T_V_ *vs* T_Temporal_	0.93	0.76	227	796	7.34	23.83
T_V_ *vs* T_MTL_	0.78	0.95	191	993	22.04	4.97
TV *vs* T_NEO_	0.76	0.98	185	1028	24.49	1.63
T_V_ *vs* Model	0.96	1.00	236	1040	3.67	0.48
ADNI^¥^						
T_V_ *vs* T_Temporal_	0.93	0.66	214	397	6.96	33.94
T_V_ *vs* T_MTL_	0.78	0.88	180	526	21.74	12.48
T_V_ *vs* T_NEO_	0.70	0.96	161	579	30.00	3.66
T_V_ vs Model	0.78	0.98	179	587	22.17	2.33
OASIS-3^ǂ^						
T_V_ *vs* T_Temporal_	0.82	0.70	50	260	18.03	29.54
T_V_ *vs* T_MTL_	0.66	0.89	40	329	34.43	10.84
T_V_ *vs* T_NEO_	0.66	0.95	40	352	34.43	4.61
Tv *vs* Model	0.75	0.98	46	364	24.59	1.36

† n = 1290; T_V+_ = 245, T_V−_ = 1045, T_Temporal_^+^ = 396, T_MTL_^+^ = 243, T_NEO_^+^ = 202

¥ n = 831; T_V+_ = 230, T_V−_ = 301, T_Temporal_^+^ = 362, T_MTL_^+^ = 255, T_NEO_^+^ = 183

ǂ n = 430; T_V+_ = 61, T_V−_ = 369, T_Temporal_^+^ = 131, T_MTL_^+^ = 80, T_NEO_^+^ = 57
